# Alcohol Consumption, *ALDH2* Polymorphism as Risk Factors for Upper Aerodigestive Tract Cancer Progression and Prognosis

**DOI:** 10.3390/life12030348

**Published:** 2022-02-27

**Authors:** Che-Hong Chen, Wen-Lun Wang, Ming-Hung Hsu, Daria Mochly-Rosen

**Affiliations:** 1 Department of Chemical and Systems Biology, Stanford University School of Medicine, Stanford, CA 94305, USA; mochly@stanford.edu; 2 Department of Internal Medicine, School of Medicine, College of Medicine, I-Shou University/E-Da Hospital, Kaohsiung 84001, Taiwan; ed102807@edah.org.tw (W.-L.W.); ed108345@edah.org.tw (M.-H.H.)

**Keywords:** *ALDH2*, acetaldehyde, cancer, upper aerodigestive tract

## Abstract

The upper aerodigestive tract (UADT) is highly susceptible to multiple primary cancers originated from squamous epithelia and constitutes a field of cancerization. Patients with head and neck cancer (head and neck squamous cell carcinoma, HNSCC) are at high risk of developing multiple cancers in the esophagus (esophageal squamous cell carcinoma, ESCC). Conversely, esophageal cancer patients are prone to develop multiple primary tumors in the head and neck region. The East Asian-specific dysfunctional ALDH2*2 missense mutation is a genetic risk factor for UADT cancer. It is not only associated with increased incidences of UADT cancer, but is also implicated in faster cancer progression and poorer prognosis. Alcohol use is a major lifestyle risk factor which causes UADT cancer among ALDH2*2 carriers. The accumulation of the immediate metabolite of alcohol, acetaldehyde, is likely the genotoxic agents that is involved in the process of tumorigenesis. This review summarizes recent publications on the risk and association of ALDH2*2 mutation, alcohol consumption in synchronous, metachronous UADT cancer. Possible molecular mechanisms involved in cancer initiation, progress and prognosis are discussed. The review also highlights a need for precision medicine-based preventive and therapeutic strategies by integrating lifestyle and genetic risk factors, such as alcohol consumption, genotypes of the alcohol metabolizing genes, *ADH1B* and *ALDH2*, into a risk assessment model for better screening, surveillance and treatment outcome.

## 1. Introduction

The upper aerodigestive tract (UADT) region, which includes the oral cavity, pharynx, larynx and esophagus, is highly susceptible to multiple primary cancers originated from squamous epithelia. This “field cancerization” in the UADT was first proposed by Slaughter et al., in 1953, to describe multiple squamous epithelial neoplastic lesions in the oral mucosa and upper respiratory tract [1]. Indeed, an analysis of 783 oral cancer patients in a U.S. hospital demonstrates that 11.2% of the patients have two or more independent squamous cell carcinoma in different locations of lips, oral cavity, larynx, pharynx, trachea, and esophagus. This finding implies that there was a regional carcinogenic activity, in which a preconditioned epithelium has been activated over an area where multiple cells undergo a process of irreversible transformation toward cancer [1]. The carcinogenic agent(s) and genetic factor(s) that exerted their effects on this epithelial field of cancerization was unknown at the time. It is now clear that exposure to lifestyle factors such as alcohol drinking, cigarette smoking, betel nut chewing, poor oral hygiene, lack of green vegetable, fruit intake and genetic factors of the alcohol metabolizing variants, *ALDH2* rs671 and *ADH1B* rs1229984, synergistically contribute to the risk of multiple squamous cell carcinoma (SCC) in UADT tract [2,3,4,5]. 

Human mitochondria aldehyde dehydrogenase 2 (*ALDH2*) is best known for its role in alcohol metabolism. Ingested alcohol is first metabolized to acetaldehyde by alcohol dehydrogenase (ADH), and acetaldehyde is further detoxified to acetate by aldehyde dehydrogenase (ALDH) in the body. The intermediate metabolite of alcohol, acetaldehyde, is a potent cytotoxic and genotoxic agent due to it reactivity with a wide range of cellular components including proteins and DNA [6]. Both alcoholic beverage and acetaldehyde associated with alcohol consumption have been designated as Group 1 human carcinogens by WHO [7,8]. Several human ADH and ALDH isozymes participate in the detoxification pathway of alcohol. However, the mitochondria *ALDH2* is the most important enzyme in acetaldehyde detoxification due to its preference (low Km) for this substrate [9]. *ALDH2* also participates in the metabolism of other cellular toxic aldehydes and oxidative stress-generated aldehydes that are also associated with human diseases [10,11]. 

Genetic polymorphisms in alcohol metabolism are common in human populations. For example, in the alcohol dehydrogenase *ADH1B* gene, an ADH1B*2 allele (rs1229984, A or H48) which is carried by the majority of the East Asians encodes a more active ADH enzyme and converts alcohol to acetaldehyde much faster than the enzyme encoded by the ADH1B*1 allele (rs1229984, G or R48) which is the predominant variant found among the Caucasians [12]. The normal *ALDH2* allele has been designated as the ALDH2*1 allele (rs671, G or E504). However, a common dysfunctional missense variant of *ALDH2*, the ALDH2*2 allele (rs671 A or K504) also exists which causes a dramatic loss of *ALDH2* activity, resulting in elevated acetaldehyde concentration after alcohol drinking [13,14]. Acetaldehyde accumulation leads to the well-known symptoms of alcohol facial flushing with concomitant acute reactions of palpitation, headache, and vomiting after alcohol consumption [15]. The low-activity ALDH2*2 missense mutation originated in East Asia approximately 2000–3000 years ago [16]. It has become a predominant genetic variant in the region affecting an estimated 8% (or 540 million) of the current world population [15]. The prevalence of ALDH2*2 carriers in the East Asian countries, such as China, Japan, Korea, and Taiwan, ranges from 30% up to 49% (or allele frequencies from 16.4% to 28.6% based on the Korean KRGDB and Taiwan Biobank database, respectively). We identified other less common *ALDH2* missense variants in different ethnic groups in the global human genome database (gnomAD, https://gnomad.broadinstitute.org, accessed on 26 February 2022). Chen et al., compiled and characterized several of these non-East Asian common *ALDH2* variants in vitro [17,18]. These variants exhibit a significant reduction in *ALDH2* enzyme activity as compared with the common (wild type) ALDH2*1 enzyme. For examples, two *ALDH2* missense variants, P92T and V304M were found with an allele frequency of 2.5% and 2.4%, respectively, in the Latino populations; these two missense variants exhibited 32% and 11% of ALDH2*1 enzyme activity in vitro, respectively. Therefore, we estimated that, in addition to the 540 million East Asians, another ~120 million non-East Asians may also have reduced *ALDH2* enzyme activity and vulnerable to acetaldehyde toxicity [17]. However, the clinical consequences or cancer risk to carriers of these additional *ALDH2* variants is currently unknown. 

Globally, 4.1% of all newly diagnosed cancer cases were attributable to alcohol consumption in 2020 [19]. Alcohol consumption has been shown unequivocally to cause cancers of the UADT, lung, S due to acetaldehyde toxicity [7,20]. For example, the relative risk of breast cancer increases by about 10% with increasing alcohol intake of every 10 g per day [21,22]. Subjects with ALDH2*2 enzyme deficiency carries are at an alarmingly higher risk especially for UADT cancers associated with alcoholic beverages consumption, even with moderate consumption (two drinks in men and one drink in women per day; one standard drink = 14 g of alcohol) [15,20]. 

Esophageal cancer tract can be classified histologically into two subtypes: esophageal squamous cell carcinoma (ESCC) and esophageal adenocarcinoma (EAC) [23,24]. ESCC, which most frequently occurs in the upper and middle third of the esophagus, is the predominant esophageal cancer in East Asia countries and highly associated with alcohol and tobacco use. In contrast, EAC, which mainly occurs in the lower third and at the gastro-esophageal junction of the digestive tract, is the predominant subtype in Western countries associated with gastroesophageal reflux disease, Barrett’s esophagus, obesity and tobacco use, but not with alcohol use directly even at high dose [25]. East Asia has the highest population-attributable fraction of cancer caused by alcohol use, especially upper esophageal cancer (ESCC) [19]. This is partly due to the high prevalence of the ALDH2*2 variant and a rapid increase in alcohol consumption in this region in the past three decades [26]. Cigarette smoking also generates elevated concentrations of acetaldehyde and other toxic aldehydes in the saliva; smoking and alcohol drinking together has been shown to lead to a seven-fold increase in saliva acetaldehyde concentration relative to drinking alone [27]. Alcohol drinking and cigarette smoking plays a slightly different role in tumor initiation and transformation. One study in Taiwan showed that smoking and betel nut chewing were more associated with the initiation of precancerous leukoplakia in oral cancer, whereas alcohol drinking was the critical factor that led to malignant transformation to cancer [28]. The strong association between ALDH2*2 mutation and the risk of UADT cancer with alcohol consumption and cigarette smoking has been reviewed in many publications [15,20,29,30,31,32]. However, less is known about the role or effect of *ALDH2* deficiency in cancer progression and prognosis. This review will therefore focus on studies that have indicated or implicated differences in clinical diagnosis, progression, prognosis and outcome in East Asian UADT cancer patients with regard to the *ALDH2* genetic status.

## 2. Synchronous and Metachronous UADT Cancer

UADT cancer patients usually have multiple primary squamous cancer lesions in different locations in the upper aerodigestive tract at or within 6 months of the first diagnosis (synchronous SCC), or in subsequent follow-ups after 6 months (metachronous SCC). For example, in Japan, routine screening by endoscopy combined with oropharyngolaryngeal inspection and esophageal iodine (Lugol voiding) staining revealed a high prevalence of multiple SCC in the upper aerodigestive tract. Based on Japan’s National Cancer Center Hospital data for esophageal cancer, the prevalence of multiple primary cancers in patients undergoing surgery increased from 6% in 1969–1980, to 22% in 1981–1991, and 39% in 1992–1996 [33], whereas the rate of synchronous and metachronous multiple cancers were only about 2–3% in the U.S. during the period 1973–2003 [34]. Head and neck SCC with synchronous SCC in the esophagus were found to occur in 8–12% of the patients in German, Brazil and Thailand [35,36], whereas in countries such as Japan and Taiwan, where ALDH2*2 are prevalent, this number has increased to 14% and 22%, respectively [37,38]. Alcohol drinking, cigarette smoking and family history are the three common risk factors for multiple UADT cancer in Japan [32]. The odds ratio (ORs) of having a second cancer in the field of UADT were 5.3 and 14.7 for smokers and drinkers, respectively, and family history was also associated with an eight-fold increase in the incidence of a second cancer based on a study in Japan [39]. Wang et al. performed endoscopic esophageal screening with image-enhanced endoscopy using narrow band image (NBI) and Lugol chromoendoscopy on 315 patients with head and neck SCC in Taiwan and reported 21.9% of the patients with synchronous esophageal neoplasia [38]. The location of oropharyngeal and hypopharyngeal cancer and habit of alcohol consumption were independent risk factors for synchronous esophageal neoplasia [38]. Furthermore, the risk of synchronous esophageal neoplasia among HNSCC patients was dependent on alcohol dose and alcohol drinking frequency (cumulative amount of alcohol exposure) [38]. A similar study on 815 head and neck cancer patients in Taiwan also showed that 15.2% of the patients had synchronous esophageal squamous-cell neoplasia [40]. There was no association between synchronous cancer and smoking or betel nut use; however, alcohol was clearly associated with synchronous cancer in this study. Even though the study did not provide direct *ALDH2* genotype information, based on an alcohol flushing questionnaire, it showed that heavy drinkers with an alcohol flushing response were 16.9 times more likely to have high-grade esophageal dysplasia and ESCC than non-drinkers. This subgroup of alcohol flushers also had the lowest overall survival among HNSCC patients [40]. 

Massive screening for oral cancer prevention has been provided by the government at no cost for qualified high-risk individuals (i.e., smokers and betel nut chewers) in Taiwan since 2004 [41]. Based on data compiled from >2.3 million oral exams between 2004–2009, it was shown that patients with head and neck cancer in the locations of hypopharynx, floor of mouth, and hard palate, but not in tongue or gum, had a greater chance of developing secondary primary cancer in the hypopharynx and esophagus. In addition, alcohol drinkers were at higher risks for second primary cancer in these two regions than non-alcohol drinkers [42]. Chien et al. examined 659 male patients with head and neck cancers in Taiwan over a period of 20 years with a minimum of 2-year follow ups and a mean follow-up period of 47.13 months for the patients [43]. In this study, 24.8% (164/659) of the HNSCC patients had multiple primary tumors within the follow-up period. The prevalence of both ALDH2*2 (rs617, A allele) and ADH1B*1 (rs1229984, G allele) were higher in the group of HNSCC patients who have developed multiple primary tumors. In addition, this HNSCC subgroup also had a significantly higher risk of developing multiple primary tumors in the upper digestive tract (OR 5.186 for ALDH2*2 and 2.093 for ADH1B*1); 84% of the HNSCC patients with multiple primary tumors in esophagus or upper GI tract had a least one ALDH2*2 allele [43].

Multiple Lugol-voiding lesions (LVLs) from Lugol chromoendoscopy are associated with exceedingly high risk of multiple cancers arising in the esophagus and the head and neck. In Japan, from a group of 76 ESCC patients, Katada et al. identified alcohol use, smoking, male, and ALDH2*2 as the risk factors associated with LVLs [44]. Alarmingly, all 36 patients diagnosed with LVL were alcohol drinkers and the proportion of patients carrying the ALDH2*2 allele increased from 67% in ESCC patients to 83% in ESCC patients with multiple lesions. With regard to the amount of alcohol consumption, patients with an ALDH2*2 allele who consumed ≥100 g alcohol per day had the highest risk of LVLs (OR 17.5) compared with ALDH2*2 patients consumed <100 g alcohol per day (OR 8.85) which was still significantly higher than patients with the ALDH2*1 normal allele but consumed ≥100 g alcohol per day (OR 4.0) [44]. Therefore, ALDH2*2 genotype greatly correlated with increased risk of multiple cancers and at a much lower threshold of alcohol consumption. Similarly, ALDH2*2, alcohol drinking and smoking were also associated with high incidents of HNSCC in patients with ESCC; Katada et al. screened 71 esophageal cancer patients and found that 29.5% of the ESCC patients also had HNSCC [45] and 95% of these patients carried the ALDH2*2 allele, 90% were also smokers and 100% of these patients consumed alcohol [45].

Metachronous ESCC is often found in patients during endoscopic treatment in Japan [46,47,48]. ALDH2*2 carriers also correlated with a greater risk of having metachronous cancer in ESCC patients. Urabe et al., identified ALDH2*2, ADH1B*1 (rs1229984, G allele) and smoking as independent risk factors for the development of metachronous squamous cell carcinoma (SCC) in a ≥12-month follow-up study on patients who had undergone endoscopic resection [49]. Having the genetic risk factors of ALDH2*1*2 and ADH1B*1*1 and lifestyle risk factor of smoking cumulatively increase the risk of metachronous SCC by 12 folds. In another study with longer follow-ups of >30 months, five independent risk factors were identified as predictors of metachronous squamous cell carcinoma development in ESCC patients after endoscopic resection [50]. The five risk factors were ALDH2*2, ADH1B*1, alcohol consumption (>20g/day), smoking, and multiple LVLs. In a cohort of 130 ESCC patients, subjects with ≥3 risk factors had significantly higher risk of developing metachronous SCC than subjects with ≤2 risk factors [50]. Construction of such a risk-factor-based appraisal model including the alcohol metabolizing genes *ALDH2*, *ADH1B* and behavioral factors of alcohol consumption and smoking could be useful for the evaluation and prediction of multiple metachronous SCC in UADT cancer patients in the future. In Japan, using a questionnaire of alcohol flushing, alcohol consumption, smoking and two dietary lifestyle factors, a risk appraisal model was successfully adopted in pilot studies of massive endoscopic screening to prevent ESCC [51,52,53]. A 2-year study involving 2221 Japanese men >50 years old showed that the detection rate of esophageal cancer was much improved and cost effective among the defined high-risk group as compared to the general patient group (4.27% vs. 0.67%) [54]. 

## 3. *ALDH2* and Cancer Prognosis

Carrying the dysfunctional ALDH2*2 variant not only increases the risk of multiple tumors among UADT cancer patients, but also leads to a poorer prognosis of these patients [55,56,57,58]. Among head and neck cancer patients, the 5-year survival rate of having a second primary caner in the head and neck region was 58%, relative to 12% and 6% with a secondary primary cancer in lung and esophagus, respectively [59]. In Japan, HPV-negative oropharyngeal cancer patients with the *ALDH2* heterozygous genotype had significantly poorer 3-year overall survival rate and disease-specific survival rate than the *ALDH2* homozygous genotypes [55]. ALDH2*2 allele, and heavy alcohol drinking were both independent prognostic factors and resulted in significantly worse 5-year overall survival and disease-free survival among 85 Japanese male hypopharyngeal squamous cell carcinoma patients [56]. In that latter study, *ADH1B* was not associated with either overall survival or disease-free survival of the hypopharyngeal SCC patients. In a larger and more recent study, Lee et al. surveyed 740 Taiwanese head and neck cancer patients with a median follow-up period of 3.1 years and reported that poorer overall survival was associated with pre-diagnosis alcohol use in a dose-dependent manner. This poorer overall survival was mainly due to its association with a higher cancer stage at the time of diagnosis. Importantly, both the dysfunctional ALDH2*2 and the fast *ADH1B* alleles were associated with the worst overall survival in HNSCC patients [57]. These studies indicate that ALDH2*2 is not only a risk factor for the incidence of UADT cancer but is also a prognostic biomarker for UADT cancer patients. 

Transcriptome analysis using bioinformatic datasets of microarray and next generation RNA-sequencing have been used widely in recent years for human cancer research. Chang et al. used publicly available data from The Cancer Genome Atlas (TCGA, https://cancergenome.nih.gov/, accessed on 26 February 2022) and Prognoscan (http://www.abren.net/PrognoScan/, accessed on 26 February 2022) databases, that contain more than 70 microarray studies of 13 different human cancer types, to examine the expression of 19 different ALDH isozymes and cancer prognosis [58]. Lower expression of *ALDH2* was consistently observed in tumor tissue samples compared to normal tissues in five different cancer types including breast cancer, lung SCC, lung adenocarcinoma, head and neck SCC and esophageal SCC. Furthermore, lower expression of *ALDH2* in tumors was associated with poorer overall survival and poorer progression-free survival [58]. Although the TCGA cohort represents data from mostly non-Asian subjects, similar results were confirmed using a smaller cohort of pairwise (tumor vs. normal) samples derived from local Taiwanese head and neck patients [58]. Interestingly, when the *ALDH2* rs671 genotype was determined in this study it was found that lower *ALDH2* expression level in the tumor was independent of the ALDH2*2 genotype. The role of *ALDH2* in tumorigenesis and progression of other cancers such as colorectal cancer, gastric cancer, liver cancer, pancreatic cancer and lung cancer has also been reviewed recently [60]. Based on this review, the expression level of *ALDH2* did not always correlate with the progression in different cancer types. The function of the inactive ALDH2*2 missense mutation and its interplay with its transcriptional level and post-translational regulation in different types of cancer progression and prognosis remains to be determined.

## 4. *ALDH2*, DNA Repair Mechanism and Somatic Mutation 

Although a direct causal link been alcohol drinking and cancer has now been established [20], alcohol by itself is not a carcinogen, whereas its immediate metabolite, acetaldehyde, is genotoxic and a proven Group 1 human carcinogen [8,61]. Acetaldehyde is a strong electrophilic compound and can react directly with DNA, causing different forms of damage including DNA adducts, single- and double-strand breaks, sister chromatid exchange and mutations [62]. Elevated acetaldehyde-DNA adducts on the deoxyguanosine moiety, N^2^-ethylidene-2′-deoxyguanosine (N^2^-ethylidene-dG), has been detected in the blood of both Japanese alcoholic patients [63] and European alcohol drinkers [64]. It is also detected in oral cells from alcohol drinkers [65], and in the esophagus of mice after ethanol administration [66]. The level of N^2^-ethylidene-dG was higher in ALDH2*2 human subjects who consumed alcohol and in *ALDH2* knockout mice as compared to ALDH2*1 human subject and *ALDH2* wild type mice [63,66]. N^2^-ethylidene-dG has been used as a biomarker to evaluate the effects of alcohol consumption on DNA damage [64] and acetaldehyde exposure derived from alcohol drinking results in a greater extent of DNA damage among ALDH2*2 subject. 

DNA repair pathway plays a key role in clearing and preventing the accumulation of acetaldehyde-mediated DNA damage. Using a combination of biochemical and bioinformatic approach, it was shown that *ALDH2* expression was negatively correlated with the level of DNA base excision repair protein, XRCC1, across several cancer types [67]. Low expression of *ALDH2* was associated with high level of XRCC1 and was indicative of poorer 5-year overall survival in liver and lung cancer patients. It was hypothesized that tumor cells growth requires robust and dysregulated single-strand break repair activity for their growth to benefit from reduced capacity of aldehyde detoxification. However, this association was not as clear in esophageal patients [67]. Another important DNA repair pathway involved with acetaldehyde-DNA adduct and interstrand cross-link repair is the Fanconi Anemia (FA) pathway. The FA pathway is composed of at least 22 FANC gene products to carry out the surveillance and repair of DNA replication stress and maintain genomic stability [68]. In recent years, acetaldehyde- and formaldehyde-toxicity have been identified as key reactive aldehyde stresses that triggers the depletion of hemopoietic stem cells, bone marrow failure, leukemia and solid tumors in FA patients [68,69]. Cells deficient in FA gene function showed chromosomal aberrations in response to acetaldehyde [70,71]. In a mouse model of FA, FANCD2 and *ALDH2* double knockout mice were hypersensitive to alcohol exposure in utero, and alcohol treatment induced a dramatic loss of borrow marrow cells in adult FANCD2 and *ALDH2* double knockout mice. These mice also developed leukemia easily even in the absence of alcohol administration [72]. 

In human, ALDH2*2 genotype has been associated with an increased risk of sporadic aplastic anemia and worse prognosis in children with idiopathic aplastic anemia [73]. Furthermore, Japanese children diagnosed with Fanconi anemia progressed more rapidly to bone marrow failure; the median time to the development of aplastic anemia was 72 months after birth in patients with homozygous wild-type ALDH2*1 genotype, relative to only 28 months in heterozygous ALDH2*1/*2 genotype, and 0 months (range 0–7) in homozygous ALDH2*2 genotype [74,75]. These studies highlighted the sensitive and susceptibility of DNA damage to aldehyde stress and the crucial role of *ALDH2* in maintaining genomic stability. Interestingly, patients with FA are not only predisposed to borrow marrow failure, acute myeloid leukemia and developmental abnormality, but are also highly vulnerable to the occurrence of solid tumors, with the most frequent solid tumor types being UADT cancers and of gynecological cancers (such as vulva carcinoma) [76]. Compared to the general population, the risk of head and neck cancer and esophageal cancer among FA patients is several hundred- to several thousand-fold higher; the estimated cumulative probability of development of a solid tumor in FA patients is 76% by the age of 45 years [76] and the hazard for these solid tumor is 8%/year [77]. The mechanism and risk factors for such a high incidence of head and neck cancer and esophageal cancer, similar to the common alcohol-induced UADT cancer, in FA patients is currently unknow. The influence of *ALDH2* genotype and aldehyde toxicity in the development, progression, and prognosis of UADT cancer in FA patients. 

Much effort in recent years has been focused on identifying genetic mutations in the tumors of ESCC patients, especially the East Asian patient cohorts; using whole-exome sequencing and single nucleotide polymorphism assay profiling, the Cancer Genome Atlas Research Network group compared mutational spectrum of somatic and germline mutations from a large number of ESCC and EAC cancer samples [78]. TP53, NFE2L2, MLLL2, ZNF750, NOTCH1 and TGFBR2 were consistently identified as the most frequent recurring mutations and SOX2, TERT, FGFR1, MDM2, NKZ2-1 and RB1 were genes with the most frequent somatic copy number amplification and deletion. These unique mutational signatures resembled HNSCC and lung LSCC and were distinct from esophageal adenocarcinoma (EAC). Among these genetic aberrations, mutations in NFE2L2 were associated with poor prognosis and resistant to chemoradiotherapy in Asian patients with ESCC [78,79]. A more recent whole exome sequencing and multi-omics study comparing Japanese ESCC and EAC patient samples also confirmed that TP53 was the most common driver mutation with somatic mutations detected in 88.6% and 62.5% of the ESCC and EAC patient tumor tissue, respectively [80]. In addition, NFE2L2, CDKN2A and KMT2D were more frequently detected in ESCC than in EAC samples. ESCC patients with FAT2 and PTPRD mutations had poor overall survival and poor progression-free survival. Importantly, as high as 79.5% of the ESCC patients were with the ALDH2*2 genotype and had enriched *ALDH2* associated Catalogue of Somatic Mutations in Cancer (COSMIC) mutational Signature 16 (i.e., increased T>C transitions) [80]. The ALDH2*2-associated mutational signature 16 was also observed in genome-wide sequence landscape of Chinese ESCC patients; the highest mutational number of Signature 16 was found in patients who were ALDH2*2 (G/A, A/A) drinkers followed by ALDH2*1 (G/G) drinkers, ALDH2*2 (G/A, A/A) non-drinkers and ALDH2*2 (GG) non-drinkers [81]. Signature 16 mutations are known to show bias toward the transcribed DNA strand and is believed to be caused by the differences in repair and maintenance of DNA damage between the transcribed and untranscribed strand of the gene [82]. These findings imply that the ESCC tumors may have inherited differences in their mutational load, repaired capacity and metastatic capacity based on the status of *ALDH2* and alcohol drinking habit of the patients. Further research is needed to elucidate the relationship between *ALDH2*, aldehyde toxicity, genome stability and solid tumor development. 

## 5. Summary

The East Asian-specific ALDH2*2 missense mutation is a genetic risk factor for UADT cancer. It is an example of interaction between strong genetic risk factors and multiple behavioral risk factors (alcohol drinking, betel nut chewing and cigarette smoking) that synergistically leads to initiation and malignant transformation to cancer. The upper aerodigestive tract is a well-defined “field cancerization”. East Asian patients with head and neck cancer (HNSCC) are at high risk of developing multiple cancers in the esophagus (ESCC). Conversely, esophageal cancer (ESCC) patients are prone to develop multiple primary tumors in the head and neck region (HNSCC). Epidemiological studies have clearly indicated that ALDH2*2 is associated with increased the susceptibility of synchronous and metachronous UADT cancers and is highly represented in the majority of these cancer patients. These patients also manifest a faster cancer progression and poor prognosis. DNA damage incurred by acetaldehyde-DNA adducts leads to mutations and genomic instability which are the likely molecular mechanism driving tumor initiation and progression in the field of cancerization. Whole genome sequencing and bioinformatics have shed new lights on the landscape of somatic mutations and specific signatures in East Asian UADT cancer patients. These methods and information can be applied to ALDH2*2 patients with multiple UADT cancer in the future for personalized medicine. Synchronous and metachronous UADT cancer also poses a challenge on surgical procedure, effectiveness of chemo- and radiation therapy and long-term survival of the patients. Since the ALDH2*2 variant allele is extremely common in East Asian countries, it highlights the importance and feasibility of new preventive strategies which should include both lifestyle and other genetic risk factors, such as *ADH1B*, in a risk assessment model for enhanced screening, patient surveillance and follow-ups. Due to its worse prognostic impact, the influence of *ALDH2* on cancer progress and prognosis, it also implies the need of knowing the genetic status of the patients and patient education when considering treatment options. Preventive strategies to reduce or mitigate local acetaldehyde exposure and acetaldehyde-DNA adduct formation can also be considered especially for high-risk ALDH2*2 individuals who are prone to have primary or multiple UADT tumors. The role of *ALDH2* deficiency in cancer progression, metastasis, prognosis and clinical treatment outcome in other cancer that not directly related to alcohol can also be the focus of future research.
